# Development and implementation of a national mobile health application: A case study from Brunei

**DOI:** 10.7189/jogh.12.03083

**Published:** 2022-12-21

**Authors:** Kai Shing Koh, Hong Shen Lim, Jeremy Lim, Yuan Wei, Phyo Wai Minn, Justin Wong

**Affiliations:** 1 Disease Control Division, Ministry of Health, Brunei Darussalam; 2 EVYD Technology Limited, Singapore; 3 Yong Yoo Lin School of Medicine, National University of Singapore, Singapore; 4 Saw Swee Hock School of Public Health, National University of Singapore, Singapore; 5 PAPRSB Institute of Health Sciences, Universiti Brunei Darussalam, Brunei Darussalam

Mobile health applications (m-Health apps) have the potential to provide low-cost, near instant access to health care to end users on a national level. They provide opportunities for remote monitoring, teleconsultation, virtual care, information exchange, and remote data capturing. National m-Health apps enable centralisation and consolidation of health data, which may assist policy and health service delivery, and allow for a continuum of care across health services. Despite the obvious potential, and the large number of m-Health apps available globally, many have not lived up to their full potential due to multiple barriers, including lack of regulatory oversight, limited user uptake, and concerns of privacy and security [1].

The SARS-CoV-2 (COVID-19) outbreak forcibly tested the preparedness and response capacities of national governments and prompted widespread adoption of digital health tools, including the implementation of m-health apps to support automated contact tracing [2,3]. In many ways, progress and scale-up models that would ordinarily take years were achieved in mere months. As countries transition towards an endemic strategy towards COVID-19, many national m-Health apps, introduced to augment a suppression strategy have fallen into disuse [4]. We believe this represents a lost opportunity to capitalize on digital models of delivering population health outcomes.

Here, we provide an example for how a national health app initially developed for COVID-19 can be adapted to support wider public health needs. BruHealth is the national m-Health app for Brunei Darussalam (population 430 000, spread across a total land area of 5770 km^2^ with GDP per capita of almost US$32 000). Initially introduced as a contact tracing tool for the national COVID-19 response, it has now broadened to integrate various health care functions including both clinical and population health management features onto a single platform.

## DEVELOPMENT OF BRUHEALTH

Brunei Darussalam reported its first COVID-19 case on March 9, 2020. Assessed against key epidemiological parameters, its subsequent response can be compared favourably to many countries, with a low case fatality ratio, and a highly vaccinated population [4]. The government has credited part of its success in this regard to the use of the BruHealth mobile application (“BruHealth”) – a digital application developed initially as a contact tracing tool.

BruHealth was launched in May 2020. At the time, the app’s main purpose was to provide a virtual platform to control the spread of COVID-19 in Brunei by focusing on contact tracing. The introduction of BruHealth required all local establishments, institutions, as well as places of worship to apply unique QR codes at entrances and exits for visitors to scan. Several additional features have since been made available including Bluetooth contact tracing, a personal health code to reflect COVID-19 infection status, issuing Digital Quarantine Orders, and automated triage for COVID-19 patients on home recovery.

**Figure:**
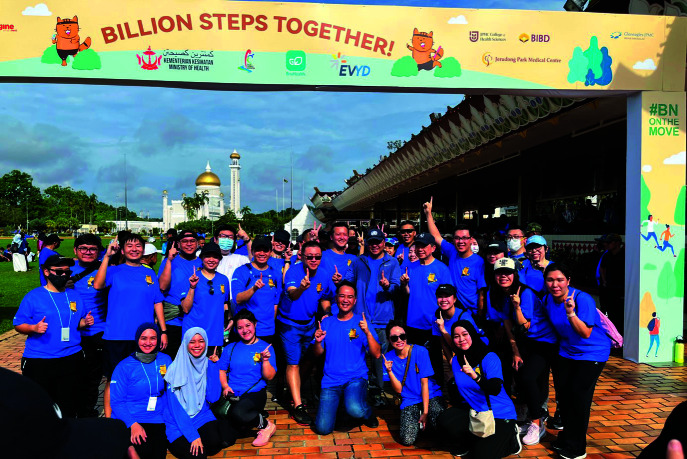
Photo: The Minister of Health and BruHealth app developers at the launch event for “BN On The Move”, a national steps challenge using BruHealth to promote physical activity in the community. Source: Wei Y.

The rollout of the National COVID-19 vaccination programme further increased the usage of BruHealth. The public were able to use it as a platform for booking vaccination appointments and to report any adverse events post-vaccination. In addition, from November 2021 up until April 2022, vaccine-differentiated measures were in place in Brunei and the app functioned as a “domestic vaccine passport” for entering buildings and public premises by reflecting the individual’s vaccination status [5].

To date, 437 711 users aged 18 years and above have downloaded and registered with BruHealth. This is larger than the population of Brunei as it includes inbound travellers and short-term visitors. As of May 10, 2022, 63% of the total resident population login to their BruHealth weekly. BruHealth has also been actively utilised for non-COVID related matters, with the most frequently accessed functions being laboratory test viewing (a module that allows users access to their laboratory test results; 566 403 unique visitors), followed by viewing of imaging results (335 320 unique visitors), checking of medication prescription records (7808 unique visitors), and booking online appointments (4259 unique visitors).

## CRITICAL SUCCESS FACTORS

The guiding principles outlined in the WHO Global Strategy on Digital Health 2020-2025 provided the framework for BruHealth’s implementation and early success [6].

**Figure 1:**
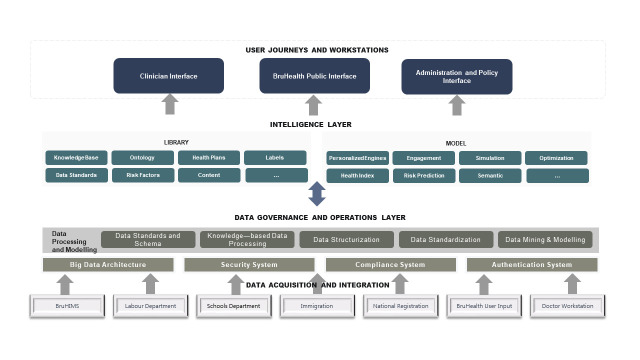
Data architecture underlying BruHealth.

First, BruHealth, while not directly inter-operable, has been integrated with the existing centralised national e-health records system, the Brunei Health Information Management System database (BruHIMS). BruHIMS covers >95% of Brunei’s resident population [7]. BruHealth has a direct application programming interface with BruHIMS enabling real-time and automated extraction of users’ health care data. The one-way syncing of health-related data from BruHIMS to BruHealth prevents tampering of existing information on BruHIMS while at the same time allows users access to view their health records via the BruHealth app. Apart from BruHIMS, details of BruHealth users are also sourced from other national databases including immigration data which provides up to date contact and address details of users, data from the Labour department which syncs information on the workplace, and data from the Department of Schools which syncs information from those registered in educational institutions (**Figure 1**). Users are also able to input their own data, including socio-demographic data, uploading COVID-19 test results, symptoms, and travel history. These data pipelines link to form the EVYDENCE platform, which is the data platform underlying BruHealth, to provide comprehensive, real-time information that continuously feeds into the BruHealth user interface to ensure records are up to date. The strong data integration allows for a continuum of care, from the clinician accessed back-end (BruHIMS) to the user accessed front-end (BruHealth) and avoids fragmentation of the digital health infrastructure in the country.

Second, BruHealth was supported by a strong public communications campaign led by the central government. In Brunei, public engagement and confidence have been achieved by active and frequent communication on BruHealth via press releases and initially near-daily press conferences by the Minister of Health and the Prime Minister’s Office. The Ministry of Health was transparent with regards to app glitches or issues and provided a platform for the public to enquire or raise complaints on any BruHealth-related problems via a national telephone hotline which is available 24 hours a day. Socialising of new features or updates on the app are actively carried out via the Ministry’s social media platform and posts on “FAQs” (Frequently Asked Questions) are also frequently updated in line with any new changes.

Third, providing an enabling environment is essential for countries to implement and adopt digital health technologies. In Brunei, the urgent need to establish a stronger public health surveillance system helped push for the development of BruHealth. This was further strengthened by having the legal infrastructure for supporting widespread adoption of BruHealth such as laws requiring proof of vaccination status for entry to certain public premises. Individuals were strongly encouraged to have BruHealth installed on their phone, or a family member’s phone if they were a dependent, whenever out in public places [8]. Until June 2022, it was compulsory for premises and businesses to display their QR code for individuals to scan prior to entering. Additionally, from the app, an individual’s health status is reflected via the personal health code generated, and those without a permitted code were denied entry to public premises. The considerable reliance on BruHealth for daily movement inevitably results in high uptake and usage of the app across the country.

## BARRIERS

One area of concern is with respect to data privacy and security issues, given the extensive integration of personally identifiable databases held by the government. In Brunei Darussalam, at present there are no specific data protection legislation or statutory law to protect individuals’ personal data. Partly as a response to the introduction of BruHealth, the Authority for Info-Communications Technology Industry of Brunei Darussalam (AITI) is currently developing a new law and has drafted the Personal Data Protection Order (PDPO) to be used as a general framework for data protection for private sector organisations that are involved in collection, use, disclosure and other processing of personal data. A Public Consultation Paper (PCP) was released to the public for feedback on May 20, 2021 and key concepts related to the draft law were also shared with the public [9].

With the usage of BruHealth being adopted nationwide, certain population groups have been put at a disadvantage. This includes the elderly population who may not have ownership of smartphones or are less tech-savvy compared to the younger generation [10]. Similarly, Brunei’s significant migrant population may experience setbacks in using BruHealth with language options either in English or Malay only, which may have limited their usability and experience with the app [11]. Although there is currently no data on user experiences and access in these population groups, it is pertinent that these factors be studied to expand accessibility and ensure equity in future enhancement of BruHealth.

## FUTURE DIRECTION

**Figure 2:**
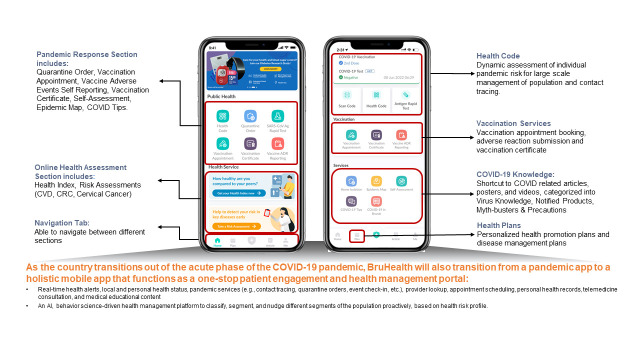
BruHealth user interface.

Since its introduction, BruHealth has shown promise as a national digital health platform. However as contact tracing capacities are scaled down and vaccine-differentiated measures removed, the use of BruHealth may gradually become redundant. To overcome this and as a way forward, the government is expanding the scope of BruHealth to be used in non-COVID-19 efforts instead (**Figure 2**).

Health promotion programmes with mobile app-based interventions have been shown to be effective in improving health promotion behaviours among the general population by providing feedback on the individual’s health status, and monitoring user’s health status or behaviour change [12]. The use of m-Health apps in managing NCDs range from monitoring of health parameters to in-app health coaching and counselling [13].

In Brunei, COVID-19 has had a negative impact on NCD control by disrupting essential health services. As part of national efforts at recovery, the government have stated its intention to use BruHealth to prioritise NCDs service expansion. This includes launching risk assessment modules for cardiovascular disease and colorectal cancer, which assists the segmentation of population for targeted NCD interventions, a health index module that updates user’s health status based on data derived from health records and self-reported data, which provides value to users through promoting health seeking behaviours, and a diabetes digital intervention. BruHealth has been recently utilised to launch a national steps challenge called “BN on the move” to encourage individuals to be active by counting or increasing their steps, and to collectively achieve 1 billion steps together as a nation – a target achieved in just 8 days, with nearly 50 000 sign-ups to the campaign during this period [14].

The COVID-19 pandemic prompted the need to strengthen public health surveillance in Brunei. BruHealth, implemented in response to this, has seen high levels of user adoption with success driven by data integration, a strong public engagement programme, and public health legislation. Initial plans to transit from a pandemic focussed app to a more holistic health app have shown promise and represents a clear opportunity for Brunei to leverage on tools built specifically for COVID-19 and test this model of digital health care.
